# Abnormal Regional Spontaneous Neural Activity and Functional Connectivity in Unmedicated Patients with Narcolepsy Type 1: A Resting-State fMRI Study

**DOI:** 10.3390/ijerph192315482

**Published:** 2022-11-22

**Authors:** Lanxiang Wu, Qingqing Zhan, Qian Liu, Suheng Xie, Sheng Tian, Liang Xie, Wei Wu

**Affiliations:** 1 Department of Neurology, The Second Affiliated Hospital of Nanchang University, Nanchang 330006, China; 2 Imaging Department, The Second Affiliated Hospital of Nanchang University, Nanchang 330006, China

**Keywords:** narcolepsy, functional magnetic resonance, fractional low-frequency amplitude of low-frequency fluctuation, connectivity, drowsiness

## Abstract

Background: Previous Resting-state functional magnetic resonance imaging (fMRI) studies have mainly focused on cerebral functional alteration in processing different emotional stimuli in patients with narcolepsy type 1 (NT1), but were short of exploration of characteristic brain activity and its remote interaction patterns. This study aimed to investigate the spontaneous blood oxygen fluctuations at rest and to elucidate the neural mechanisms underlying neuropsychiatric behavior. Method: A total of 18 unmedicated patients with NT1 and matched healthy individuals were recruited in a resting-state fMRI study. Magnetic resonance imaging (MRI) data were first analyzed using fractional low-frequency amplitude of low-frequency fluctuation (fALFF) to detect changes in local neural activity, and regions with group differences were taken as regions of interest (ROIs). Secondly, functional connectivity (FC) analysis was used to explore altered connectivity between ROIs and other areas. Lastly, the relationship between functional brain activity and neuropsychiatric behaviors was analyzed with correlation analysis. Results: fALFF analysis revealed enhanced neural activity in bilateral fusiform gyrus (FFG), right precentral gyrus, and left postcentral gyrus (PoCG) in the NT1 group. The patients indicated reduced activity in the bilateral temporal pole middle temporal gyrus (TPOmid), left caudate nucleus (CAU), left parahippocampus, left precuneus (PCUN), right amygdala, and right anterior cingulate and paracingulate gyri. ESS score was negatively correlated with fALFF in the right FFG. The NT1 group revealed decreased connectivity between left TPOmid and right PoCG, the bilateral middle frontal gyrus, left superior frontal gyrus, medial, and right supramarginal gyrus. Epworth Sleepiness Scale (ESS) was negatively correlated with FC of the left TPOmid with left putamen (PUT) in NT1. Compared with healthy controls (HCs), enhanced FC of the left CAU with right FFG was positively associated with MSLT-SOREMPs in patients. Furthermore, increased FC of the left PCUN with right PoCG was positively correlated with SDS score. Conclusions: We found that multiple functional activities related to the processing of emotional regulation and sensory information processing were abnormal, and some were related to clinical characteristics. fALFF in the left postcentral or right precentral gyrus may be used as a biomarker of narcolepsy, whereas fALFF in the right fusiform and the FC strength of the left temporal pole middle temporal gyrus with the putamen may be clinical indicators to assess the drowsiness severity of narcolepsy.

## 1. Introduction

Narcolepsy type 1 (NT1) is a rare neurological disorder, with a prevalence of only 0.02% to 0.05% [1,2]. Due to uncontrollable lethargy and unpredictable cataplexy, patients with narcolepsy are seriously affected in their ability to study, live, and work, while even causing accidents that endanger lives and property [3]. As there is no objective biomarker for this condition, in addition to a lack of knowledge of the disease among doctors, more than half of patients are misdiagnosed, sometimes for even up to 8–10 years [4]. Studies have indicated that 90% of NT1 cases are related to low or absent levels of orexin due to apoptosis in the hypothalamus neurons [5], but the exact pathogenesis underpinning narcolepsy remains elusive and no cure exists. Thus, it is imperative to detect potential biomarkers and explore the neural mechanisms as a means of improving the early diagnosis and therapeutic interventions of narcolepsy.

The fibers from hypothalamic secretin neurons project densely onto the cortical and subcortical brain areas, regulating cyclical changes in sleep and wakefulness. In NT1, the areas involved in orexin deficiency extend beyond the hypothalamus to include a wide range of the brain [6,7]. Recently, increasing neuroimaging studies have attempted to confirm the structural and functional abnormalities of the cranial brain in narcolepsy, but their results remained controversial [8,9,10,11,12]. Resting-state functional magnetic resonance imaging (fMRI) is an advanced and rapidly evolving tool, with an emphasis on identifying the neurobiological underpinnings of pathophysiologic mechanisms that are unobservable at the behavioral level, while also providing targets for future clinical interventions [13]. Considering its advantages of non-invasiveness, simplicity, and the variety of analysis methods, it has been well-established for studying various neurological diseases, including Alzheimer’s disease, Parkinson’s disease, and depression [14]. A recent investigation based on independent component and graph theoretical analysis reported aberrant connectivity in executive and salient networks in patients with narcolepsy [15]. Another analysis using the hypothalamus and amygdala as seed points revealed altered connectivity of the hypothalamus with the left superior parietal lobe and the parahippocampal gyrus, as well as of the amygdala with postcentral gyrus, insula, and nucleus accumbens [16]. Nevertheless, little is known about resting-state fMRI in narcolepsy, especially with regard to the characteristic brain regions and interaction patterns of brain functional activity. Currently, low-frequency fluctuation amplitude (ALFF) and functional connectivity (FC) analyses are efficient, widely used methods in resting-state fMRI research [13]. ALFF defines a specific frequency range of 0.01–0.08 Hz to study local brain neural activity by analyzing regional spontaneous fluctuations in BOLD signals [17]. To improve the original ALFF approach, a modified measure called fractional ALFF (fALFF) was introduced, which has been demonstrated to remove the disturbance of non-specific noise components [13]. In view of its stability and robustness, fALFF can be used as a biomarker in neuroimaging research [18]. Furthermore, data-driven FC analysis can balance the subjective differences in seed point selection to more objectively reveal correlated fluctuations in BOLD signals between discrete brain regions during rest [19]. 

To our knowledge, this study is the first to use a network-based, data-driven, whole-brain approach to explore differences in local brain activity and whole-brain functional connectivity between a cohort of NT1 patients and healthy controls, relating these alterations to neuropsychiatric behaviors. The main objective was to elucidate potential neuroimaging features and task-free brain mechanisms of narcolepsy, which may provide guidance for follow-up diagnostic and therapeutic research.

## 2. Materials and Methods

### 2.1. Participant Recruitment

This trial was approved by the institutional review board of the Second Affiliated Hospital of Nanchang University, and conducted strictly according to the ethical principles of the Declaration of Helsinki. All participants were recruited from the Sleep Medicine Center of the Neurology Department and the Health Checkup Center of the hospital from September 2019 to November 2021. After completely understanding the description of the experiment and signing the informed consent form, participants were assigned to examination. Narcolepsy type 1 was diagnosed by two sleep specialists in accordance with the International Classification of Sleep Disorders criteria (ICSD)-3 for narcolepsy [20], constituting excessive daytime sleepiness for more than 3 months and clear history of cataplexy, combining multiple sleep latency test (MSLT) results and orexin A (Hcrt-1) concentration in cerebrospinal fluid. All NT1 patients were diagnosed for the first time and not medicated with psychiatric stimulants. Moreover, participants with any of the following conditions were excluded: (1) chronic pulmonary or heart disease, diabetes, and psychosis disorder; (2) abnormality of neurological physical examination or structural lesion on cranial MRI findings; (3) drug, alcohol, and substance abuse; (4) claustrophobia, pregnancy or lactation, and other general contraindications against MRI examination. The healthy controls had neither psychiatric nor neurologic conditions, and they were negative for sleep disorders according to questionnaires and interviews prior to examination. Furthermore, all participants were right-handed and Han Chinese (Table 1).

### 2.2. Clinical Evaluation

All participants underwent an evaluation using the Epworth Sleepiness Scale (ESS), Self-rating Anxiety Scale (SAS), Self-rating Depression Scale (SDS), Pittsburgh Sleep Quality Index (PSQI), Insomnia Severity Scale (ISI), and Multidimensional Fatigue Inventory (MFI-20). They were required to abstain from smoking, coffee, and alcoholic drinks for at least 24 h prior to this research. Next, all NT1 patients conducted 8 h nighttime polysomnography and a clinical MSLT the day after the nocturnal polysomnography. Following the American Academy of Sleep Medicine Guidelines, scheduled naps were conducted every 2 h after waking up in the morning. If there was no sleep for 20 min, the nap trial ended and the sleep latency was documented as 20 min. If patients fell asleep within 20 min, the sleep latency was defined as the time from lights out to the first sleep epoch (including stage 1). The test continued for more than 15 min after being asleep to estimate the presence of REM sleep. If present, the latency of REM sleep was recorded. The mean sleep latency for five naps in MSLT (MSLT-SL) and the number of occurrences of REM sleep were then calculated (MSLT-SOREMPs).

### 2.3. Imaging Data Acquisition

After finishing the daytime MSLT, subjects were scheduled for an MRI scan using a GE Discovery MR750 3.0 T system with an eight-channel, phased-array head coil (General Electric, Milwaukee, WI, USA) in the Imaging Center of the Second Affiliated Hospital of Nanchang University. Parameters for functional images were as follows: gradient echo planar imaging sequence echo time/repetition time (TE/TR) of 35/2000 ms, 64 × 64 matrix, 4 mm slice thickness, 39 slices, 90° flip angle, field of view (FOV) of 240 × 240 mm, and 9360 images lasting 480 s. Then, a high-resolution T1 image was scanned for anatomical reference (3-D Bravo T1-weighted sequence) with the following parameters: TE/TR = 3.3/8.5 ms, 1 mm slice thickness, 90° flip angle, FOV of 240 × 240 mm, 256 images lasting 195 s. Foam padding was used to for each subject to reduce involuntary head motions. During the scanning, all participants were asked to remain awake, with eyes open, and lie motionless with no systematic thinking, supervised by both a radiologist and a technician via video. 

### 2.4. MRI Data Preprocessing

All MRI data preprocessing was conducted using the statistic parametric mapping software package (SPM12, http://www.fil.ion.ucl.ac.uk/spm (accessed on 13 January 2020)) and the Data Processing Assistant for Resting-State fMRI (DPARSF 5.1, http://rfmri.org/DPARSF (accessed on 11 October 2020)) running in MATLAB2013b (MathWorks, Natick, MA, USA). All DICOM files were first converted to NIFTI images, and the first 10 functional volume images of each participant’s dataset were discarded to allow the subjects to adapt to the scanning environment and the signal to reach equilibrium. Then, the remaining data were corrected for slice timing and realigned for motion correction, while removing the imaging data of average head motion exceeding 3 mm in translation and 3° in rotation. Anatomical and functional images were first manually reoriented to the anterior commissure, and structural images were co-registered to the functional images for each participant using a linear transformation. Then, the images were normalized to the standard Montreal Neurological Institute space template using “DARTEL + new segment” and reassembled to a voxel size of 3 × 3 × 3 mm^3^. The signals of linear drift, signals of white matter, Friston 24-parameter model, and cerebrospinal fluid were regressed as covariates from the time series of every voxel.

### 2.5. fALFF Analysis

The fALFF analysis was conducted using the DPABI V5.0 and SPM12 software. Firstly, the time series of each voxel was transformed into the frequency domain to obtain the power spectrum. Specifically, fast Fourier transform (FFT) was conducted on the time series of each voxel without temporal band-pass filtering to calculate the individual ALFF map. Then, the amplitude of each frequency was estimated by computing the square root of the power spectrum. fALFF is the ratio of the BOLD signal fluctuation in the low-frequency range relative to the entire frequency range, regarded as one of the ways to regress some of the horizontal signals of the whole brain. Moreover, the fALFF values were converted to *z*-fALFF values using Fisher’s *z*-transformation to eliminate individual heterogeneity among the subjects. The *z*-fALFF values were spatially smoothed using an anisotropic Gaussian kernel with a 4 mm full-width at half-maximum, and then used as the final index for the statistical analysis.

### 2.6. fALFF-Based Whole-Brain FC Analysis

After the fALFF analysis, the regions with statistical differences in brain activity were saved as ROIs to further investigate the integration of the brain function network via whole-brain FC analysis using DPABI V5.0 software. Firstly, the data were preprocessed using a band-pass filter and spatially smoothed with a 4 mm FWHM Gaussian kernel. Then, a voxel-wise FC analysis was performed by calculating the linear correlations between the mean time series within each ROI and the time series of each voxel across the whole brain for each participant. Lastly, the correlation coefficients (r) were normalized to *z*-scores using the Fisher r-to-*z* transformation with the following equation to create subject-specific maps:

*z* = 0.5 log [1 + r/1 − r] (1)

The *z*-score FC maps of each ROI were generated, and further statistical analysis was conducted.

### 2.7. Statistical Analysis

The differences in demographic data and clinical characteristics were computed with the independent two-sample *t*-test or the chi-square test between two groups using the IBM Statistical Package for the Social Sciences 23.0 software (IBM SPSS Inc, Chicago, IL, USA). For the fALFF-based whole-brain FC analysis, one-sample *t*-tests were first performed on individual *z*-maps to create masks for separate within-group comparisons (within gray matter masks) within each group separately. A two-sample *t*-test was applied to calculate different *z*-FC values using the generated masks. We applied a two-sample *t*-test to identify group differences in fALFF and FC, regressed for age, gender, educational level, and whole-brain gray matter volume. The result was subsequently corrected using Gaussian random field (GRF) theory. A statistical threshold of *p* < 0.001 was first applied, which is the recommended minimum height threshold. A range threshold of *p* < 0.05 was then adjusted for cluster-level multiple comparisons. In addition, after extracting the mean eigenvalues of fALFF and FC values in statistically different brain regions in the NT1 group, Pearson correlation analysis was used to investigate the correlation of abnormal fALFF and FC values with clinical indicators, regressed for age and gender.

## 3. Result

### 3.1. Demographic Data and Clinical Features

In the study, 5 out of 23 participants were excluded in view of excessive head movement over 3 mm. There were no group differences in age, gender distribution, years of education, and PSQI and ISI scores. Furthermore, the NT1 group showed higher SAS, SDS, and ESS scores than the HC group (Table 2).

### 3.2. fALFF Difference across Groups

**Figure 1:**
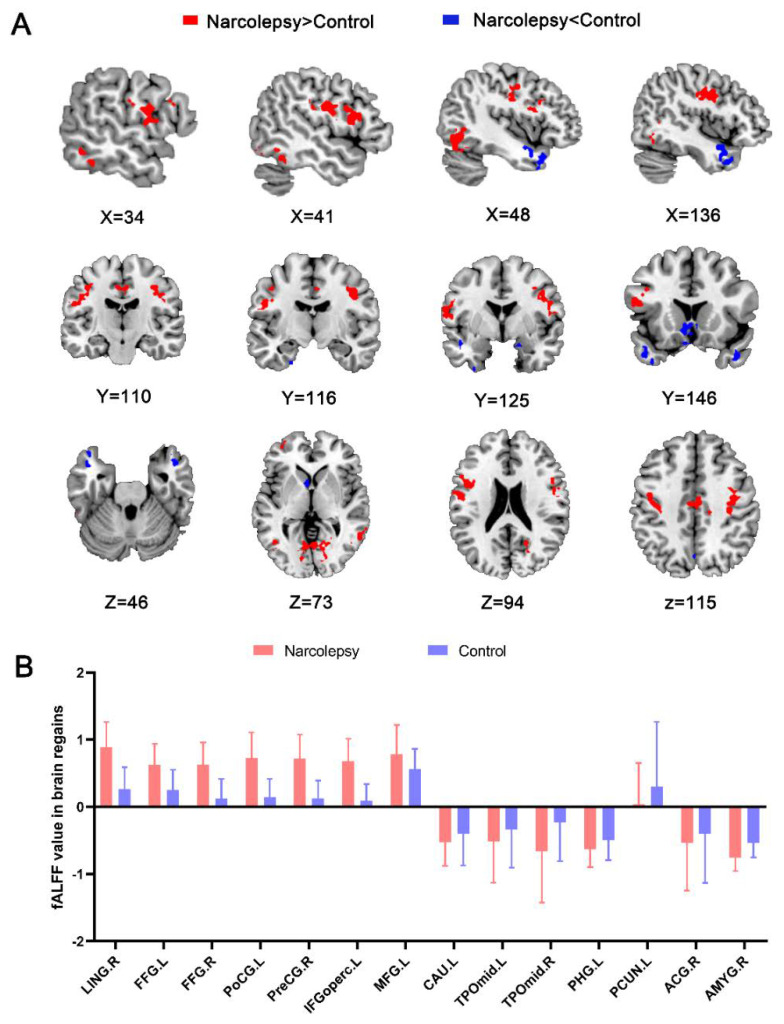
Statistical map depicts higher and lower fALFF values in the NT1 group compared with healthy controls (**A**). Red and blue denote increased and decreased fALFF, respectively (*p* voxel < 0.001, *p*-cluster < 0.05, GRF-corrected). Comparison of fALFF values in regional brain between narcolepsy and HCs (**B**). fALFF, fractional low-frequency amplitude of low-frequency fluctuation. NT1, narcolepsy type 1. LING, lingual gyrus. FFG, fusiform gyrus. PoCG, postcentral gyrus. IFGoperc, left inferior frontal gyrus, opercular part. MFG, left middle frontal gyrus. TPOmid, temporal pole, middle temporal gyrus. CAU, caudate nucleus. PHG, parahippocampal. PCUN, precuneus. ACG, anterior cingulate and paracingulate gyri. AMYG, amygdala. L, left. R, right.

Relative to the HCs, the NT1 group exhibited increased fALFF in the bilateral fusiform gyrus (FFG), left postcentral gyrus (PoCG), left inferior frontal gyrus opercular part (IFGoperc), left middle frontal gyrus (MFG), right lingual gyrus (LING), and right precentral gyri (PreCG). The patients showed decreased fALFF values in bilateral temporal pole middle temporal gyrus (TPOmid), left caudate nucleus (CAU), left precuneus (PCUN), left parahippocampus (PHG), right anterior cingulate and paracingulate gyri (ACG), and right amygdala (AMYG) (Table 3 and Figure 1A,B).

**Figure 2:**
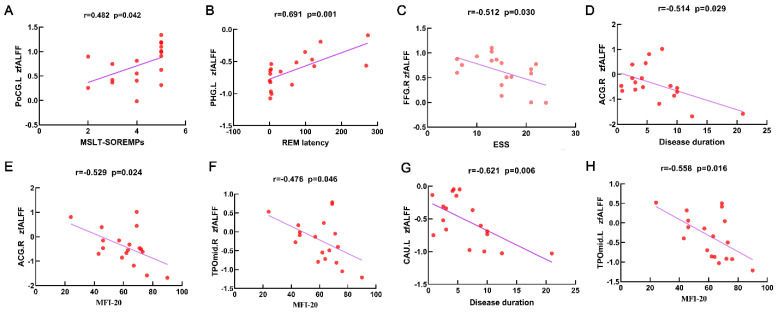
Scatter plots and correlation analysis between clinical characteristics and the fALFF in differential brain regions from the NT1 group. Scatter plots and correlation analysis between MSLT-SOREMPs and fALFF in the left PoCG (**A**). Scatter plots and correlation analysis between REM latency and fALFF in the left PHG (**B**). Scatter plots and correlation analysis between ESS and fALFF in the right FFG (**C**). Scatter plots and correlation analysis between disease duration and fALFF in the right ACG (**D**), left CAU (**G**). Scatter plots and correlation analysis between MFI-20 and fALFF in the right ACG (**E**), right TPOmid (**F**), left TPOmid (**H**). fALFF, fractional low-frequency amplitude of low-frequency fluctuation. NT1, narcolepsy type 1. MSLT-SOREMPs, the mean sleep latency for 5 naps in multiple sleep latency test. REM, rapid eye movement. ESS, Epworth Sleepiness Scale. MFI-20, Multidimensional Fatigue Inventory. PoCG, postcentral gyrus. PHG, parahippocampal. FFG, fusiform gyrus. ACG, anterior cingulate and paracingulate gyri. TPOmid, temporal pole, middle temporal gyrus. CAU, caudate nucleus. L, left. R, right.

For the NTI group, the MSLT-SOREMPs was positively correlated with fALFF value in the left PoCG (r = 0.482, *p* = 0.042, Figure 2A), REM latency in polysomnography was positively correlated with fALFF value in the left PHG (r = 0.691, *p* = 0.001, Figure 2B), and ESS was negatively correlated with fALFF value in the right FFG (r = −0.512, *p* = 0.030, Figure 2C). Furthermore, disease duration was negatively correlated with fALFF value in the right ACG (r = −0.514, *p* = 0.029, Figure 2D) and left CAU (r = −0.621, *p* = 0.006, Figure 2G), and MFI-20 was negatively correlated with fALFF value in the right ACG (r = −0.529, *p* = 0.024, Figure 2E) and bilateral TPOmid (r = −0.476/r = −0.558, *p* = 0.045/*p* = 0.016, Figure 2F,H).

### 3.3. fALFF-Based Whole-Brain FC Analysis

**Figure 3:**
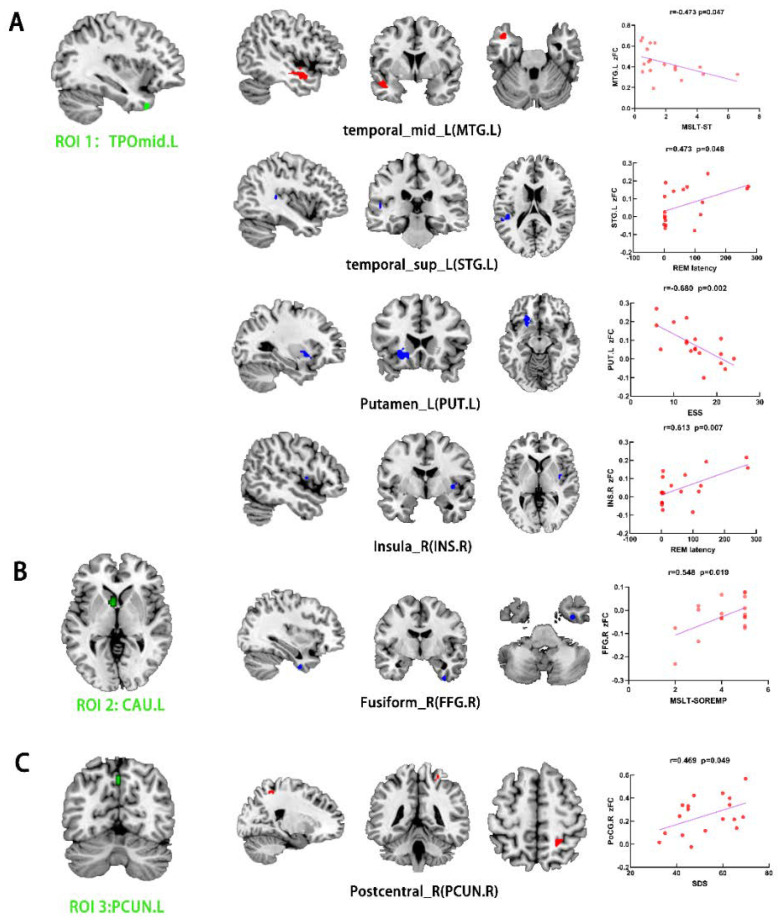
Group differences in FC analysis using ROI1 (TPOmid.L) (**A**), ROI2 (CAU.L) (**B**), and ROI3 (PCUN.L) (**C**) as seeds among narcolepsy and HC groups, respectively (*p* voxel < 0.001, *p*-cluster < 0.05, GRF-corrected). Green represents seed points for FC analysis. Red and blue denote increased and decreased FC, respectively. Scatter plots and correlation analysis between clinical characteristics and the fALFF in differential brain regions from NT1 group. ROI, region of interest. FC, functional connectivity. MSLT-SL, average RME latency in multiple sleep latency test. MSLT-SOREMPs, the mean sleep latency for 5 naps in multiple sleep latency test. ESS, Epworth Sleepiness Scale. SDS, Self-rating Depression Scale. TPOmid, temporal pole, middle temporal gyrus. CAU, caudate nucleus. PCUN, precuneus. MTG, middle temporal gyrus. STG, superior temporal gyrus. PUT, lenticular nucleus, putamen. INS, insula. FFG, fusiform gyrus. PoCG, postcentral gyrus. L, left. R, right.

Relative to the HCs, the NT1 group showed increased FC between the left TPOmid (ROI 1) and left middle temporal gyrus (MTG), as well as the right temporal pole superior temporal gyrus (TPOsup). On the other hand, the NT1 group revealed decreased FC between the left TPOmid (ROI 1) and right PoCG, the bilateral MFG, left lenticular nucleus, putamen (PUT), inferior frontal gyrus opercular part (IFGoperc), left superior temporal gyrus (STG), left superior frontal gyrus medial (SFGmed), right insula (INS), right supramarginal gyrus (SMG), and CAU (Table 4, Figure 3A). Compared with HCs, FC between the left CAU (ROI 2) with right FFG was enhanced in the NT1 group (Table 4, Figure 3B). In comparison with the HCs, increased FC between left PCUN (ROI 3) and right PoCG was observed in the NTI group. In contrast, decreased FC was observed for the right PCUN, left SFGmed, and MTG (Table 4, Figure 3C).

For the NTI group, the REM latency was positively correlated with FC of the left TPOmid with right INS (r = 0.613, *p* = 0.007, Figure 3A) and left STG (r = 0.473, *p* = 0.048, Figure 3A). The MSLT-SL was negatively correlated with FC of the left TPOmid with MTG (r = −0.473, *p* = 0.047, Figure 3A). ESS score was negatively correlated with FC value of the left TPOmid with PUT in the NT1 group (r = −0.680, *p* = 0.002, Figure 3A). Furthermore, there was a positive association between FC of the left CAU with right FFG with MSLT-SOREMPs (r = 0.548, *p* = 0.019, Figure 3B). SDS score was positively correlated with FC of the left PCUN with right PoCG (r = 0.469, *p* = 0.049, Figure 3C).

## 4. Discussion

In this study, we used data-driven FC analysis and calibration methods for the first time, to obtain some trustworthy results in narcolepsy. We found that NT1 patients had abnormalities in the local activity and connectivity at rest in multiple brain regions, mainly related to emotion regulation and sensory information processing. Moreover, differences in imaging indicators in these brain regions were correlated with clinical indicators, especially fatigue levels, depression scales, and the indices of the multiple sleep latency test and polysomnography.

In narcolepsy patients, cataplexy is sudden muscle paralysis triggered by generally positive emotions [6]. Furthermore, mood disorders have been linked to shortened nocturnal REM sleep latency, nocturnal sleep fragmentation, and episodic hallucinations in NT1 [21]. Similar to previous studies, we found NT1 patients to have high rates of depression and anxiety [2,22]. Furthermore, decreased fALFF was demonstrated in three brain regions associated with emotion regulation, namely the parahippocampus, amygdala, and anterior cingulate and paracingulate gyri. Meanwhile, several previous investigations reported gray matter reductions and inadequate cerebral perfusion in these brain regions during wakefulness [23,24,25]. These results suggest that dysfunction in emotion-regulating brain regions may be involved in the complex developmental mechanisms of narcolepsy. The fALFF in these regions had no correlation with depression and anxiety level in narcolepsy, which may be related to the heterogeneity of the small sample size; thus, further research is warranted. In addition to cataplexy, the rapid transition from wakefulness to REM sleep is a polysomnographic marker of NT1. Our findings revealed that reduced fALFF in the parahippocampus was positively correlated with the REM latency, consistent with existing theories. Studies have demonstrated that reduced volume in the hippocampus and amygdala is related to clinical features of narcolepsy [26]. In addition, modafinil, a wakefulness-promoting agent for the treatment of NT1 daytime sleepiness, is thought to protect hippocampal neurons primarily by inhibiting excessive autophagy and apoptosis [27]. Furthermore, modafinil treatment for 2 weeks can increase the metabolic level in the hippocampus [28]. It was reported that developmental disorders of hippocampal neurons may be related to the decrease in orexin secreted by the hypothalamus [29]. Therefore, changing the neuronal activity of the hippocampus may be a new direction for the management of narcolepsy. Substantial evidence has indicated that the amygdala exhibits bidirectional connections with the hypothalamus and relevant areas modulating sensory information in the temporal and insular cortex; this brain region was also implicated in REM sleep cycle regulation processes, and was thought to underlie cataplexy episodes [30,31]. Two previous studies described decreased gray matter of the amygdala in narcolepsy patients [26,32]. Moreover, neurodegeneration with gliosis was found in the amygdala [33]. In addition to the structural changes in the amygdala, the present study found reduced right amygdala neurol activity at rest, further pointing to the involvement of altered amygdala neuronal activity in the pathophysiological mechanism of NT1, which deserves further investigation. In contrast to a previous report [16], there were no alterations in amygdala connectivity in our findings, which may have been caused by bias in the method of seed point selection, whereas the data-driven approach and rigorous correction methods used in this study may have yielded more reliable results. Conversely, task-state fMRI triggered sudden catalepsy onset in 10 patients using an interesting video, which showed obvious activation in the amygdala [21]. Therefore, we speculate that altering the activation state of the amygdala may elicit catalepsy onset in NTI and may be a clinical target for preventing catalepsy onset. In summary, we believe that the onset and development of narcolepsy may be related to abnormalities in the parahippocampal gyrus and amygdala, and the specific biological mechanism deserves further investigation.

The caudate nucleus is a key node of the salience network, and modulation of functional connectivity in the salience network can alter the body’s arousal state and sleep architecture [34]. Studies have shown that caudate nucleus activity is related to the release of monoamines such as dopamine, which can participate in the regulation of cataplexy through D2-like receptors and play a role in maintaining vigilance and wakefulness [35]. Furthermore, studies have noted decreased FA values in the caudate nucleus of narcolepsy, supporting that altered functional activity in this region is involved in the pathogenesis of NT1 [36]. In our study, even though all patients were awake and alert during MRI scans, weakened left caudate nucleus activity was observed, suggesting that maintaining normal activation of the caudate nucleus may improve somnolence in NT1 patients. The present study found elevated ALFF values in the lingual gyrus and bilateral fusiform gyrus, which are associated with visual information processing, consistent with previous studies indicating high metabolism in this brain region [10]. Furthermore, connectivity of the caudate nucleus with the fusiform gyrus was positively correlated with MSLT-SOREMPs, indicating the presence of arousal facilitation dysfunction in narcolepsy, which was previously demonstrated in a pharmacological study [37]. The elevated local neurol activity in the left postcentral and the right precentral gyrus in this study is consistent with previous fALFF analysis [38]. Consistent with our results, several studies observed a bilateral pre- and postcentral gyrus with higher metabolism, higher perfusion [10,39], and an increase in apparent diffusion coefficients [40]. Moreover, blood flow in the precentral gyrus was reduced after modafinil administration [41]. These results suggest that fALFF in these two brain areas is reproducible and can be implemented as an initial screening biomarker for NT1, which should be further verified in follow-up study. We propose that increased activity in the pre- and postcentral gyri may be compensating for the shortage of hypothalamic secretin in the patient’s motor cortex, although a contradictory result report indicated the lack of sensorimotor cortical activity in NT1 via transcranial magnetic stimulation [42]. Different sample heterogeneity and scan parameters might have resulted in the discrepancy. Additionally, FC strength between the left precuneus and right precentral gyrus was found to be related to depression severity in narcolepsy, which, to our knowledge, has not previously been reported in the literature. Additionally, the precuneus and postcentral gyrus are the most commonly altered areas in depressive disorder; hence, connectivity abnormality in these two brain areas may be a mechanism via which depressed mood occurs in narcolepsy. In this study, lower fALFF in the bilateral TPOmid was indicated, inconsistent with glucose metabolism alteration in the temporal lobe in previous studies [24,43]. The temporal lobe is primarily involved in visual perception, sensory information processing, and emotional activity [44]. It has also been shown that the level of glucose metabolism in the middle temporal gyrus is negatively correlated with the time to fall asleep in NT1 patients [39].

Meanwhile, several studies demonstrated gray matter reductions in temporal cortices [25,45]. Furthermore, fALFF strength in this brain region was negatively correlated with the severity of fatigue score in patients; therefore, fALFF value in the left TPOmid could be a stable clinical index to assess the severity of fatigue in narcolepsy, which is a distinctive evolution pattern of narcolepsy’s influence on cerebral function. Several findings noted reductions in volume [46] and FA in the white matter of the frontal and temporal area in NTI patients [47]. The present study demonstrated low connectivity between the left TPOmid and frontal lobe, including the middle frontal gyrus, IFGoperc and SFGmed, indicating that abnormal frontal and temporal lobe connectivity may participate in the occurrence and developmental mechanism of NTI. In our results, the FC strength of the left TPOmid and putamen, and the fALFF value in the right fusiform were proportional to the severity of daytime sleepiness, indicating that altered FC and fALFF values in corresponding regions may be a clinical indictor to evaluate subjective hypnosis in narcolepsy. On the basis of these findings, it is reasonable to hypothesize that abnormal functional connectivity of brain regions associated with the sensory system may lead to dysfunction in visual and somatosensory processing, which in turn impedes the ability to maintain wakefulness. Therefore, the caudate nucleus, the pre- and postcentral gyri, and the temporal lobe are areas that deserve further focus in narcolepsy.

Despite our systematic findings, the present study is not free from limitations. Firstly, the sample size of was small and can be further expanded for future research. Secondly, despite being fully awake during the scanning session as controlled by video, the participants’ state was not confirmed by simultaneous electroencephalography (EEG) during the MRI scan. Thirdly, although participants were requested to clear their brains throughout the MRI scan, their mental activity could not be fully controlled.

## 5. Conclusions

We found that multiple functional activities related to the processing of emotional regulation and sensory information processing were abnormal, and some were related to clinical symptoms. The fALFF value in the left postcentral or right precentral gyrus may be a biomarker of narcolepsy. The fALFF value in the right fusiform and the FC strength of the left temporal pole middle temporal gyrus with the putamen may be clinical indicators to assess the drowsiness severity of narcolepsy.

## Figures and Tables

**Table 1:** Clinical and laboratory data of patients (included in the fMRI group analyses).

ID	Age	Sex	Disease Duration (years)	HLA-DQBI Gene	CSF Hcrt (pg/mL)	MSLT-SL	MSLT-SOREMPs
1	20.60	M	10.00	06:02,03:01	23.43	1.00	5.00
2	14.50	M	9.50	06:02,03:01	16.44	0.80	5.00
3	21.25	M	2.50	06:02,03:01	20.92	1.00	5.00
4	21.25	F	8.90	06:02,02:02	30.86	0.40	5.00
5	18.80	F	4.80	06:02,05:02	14.70	0.50	4.00
6	24.60	M	3.00	06:02,04:01	none	1.00	5.00
7	19.60	M	4.30	06:02,03:02	30.92	4.40	3.00
8	16.33	M	10.00	06:02,02:02	none	0.90	5.00
9	16.90	F	4.10	06:02,04:02	none	3.00	5.00
10	16.33	M	3.00	06:02;06:02	none	0.50	3.00
11	18.67	F	7.00	06:02;03:03	none	4.20	4.00
12	17.50	M	0.83	05:02;06:02	46.80	0.60	5.00
13	30.10	M	0.67	06:02;03:03	61.90	1.20	4.00
14	21.70	F	7.50	06:02;02:02	22.61	6.60	4.00
15	19.75	M	5.30	06:02;03:03	23.58	1.90	2.00
16	28.10	F	2.50	06:02;02:01	25.40	1.30	2.00
17	35.50	F	21.00	06:02;05:02	60.28	2.60	3.00
18	23.50	F	12.50	06:02;02:02	15.61	2.60	5.00

M, male. F, female. CSF Hcrt, orexin concentration in cerebrospinal fluid. HLA-DQBI, one kind of human leukocyte antigen. MSLT-SL, average RME latency in multiple sleep latency test. MSLT-SOREMPs, the number of occurrences of REM sleep in multiple sleep latency test.

**Table 2:** Demographic and clinical characteristics of NT1 and HC groups.

Variables	NT1	Controls	*t*/X^2^	*p* Value
Demographics				
N	18	18		
Age (years)	21.39 ± 5.37	22.30 ± 4.56	0.186	0.605 ^b^
Gender (F/M)	8/10	7/11	1.003	0.317 ^a^
Education (years)	10.83 ± 2.12	11.11 ± 2.46	0.097	0.705 ^b^
Disease duration (years)	6.52 ± 4.99	-	-	-
Questionnaire scores				
SDS scores	58.72 ± 5.62	35.60 ± 3.12	1.656	0.000 ^b^
SAS scores	45.39 ± 6.14	41.22 ± 4.68	2.496	0.028 ^b^
PSQI scores	11.56 ± 3.55	10.94 ± 4.14	0.023	0.637 ^b^
ESS scores	17.22 ± 1.73	4.35 ± 1.24	1.514	0.000 ^b^
ISI scores	13.89 ± 6.87	11.61 ± 3.87	7.250	0.229 ^b^
MFI-20	60.89 ± 15.39	-	-	-
Multiple Sleep Latency Test				
MSLT-SL	1.10(0.75,2.70)	-	-	-
MSLT-SOREMP	4.00(3.00,5.00)	-	-	-
Polysomnography				
SOL	3.00(1.25,6.13)	-	-	-
TST (minutes)	458.50 ± 79.10	-	-	-
Sleep efficiency (%)	83.25(81.61,90.86)	-	-	-
Sleep architecture	18.50(2.38,120)	-	-	-
N1%	20.20(16.65,29.70)	-	-	-
N2%	42.25(38.05,44.45)	-	-	-
N3%	18.54 ± 5.83	-	-	-
REM%	17.32 ± 6.80	-	-	-

NT1, narcolepsy type 1. HCs, healthy controls. F, female. M, male. SDS, Self-rating Depression Scale. SAS, Self-rating Anxiety Scale. PSQI, Pittsburgh Sleep Quality Index. ESS, Epworth Sleepiness Scale. ISI, Insomnia Severity Scale. MFI-20, Multidimensional Fatigue Inventory. REM, rapid eye movement. MSLT-SL, average RME latency in multiple sleep latency test. MSLT-SOREMPs, the mean sleep latency for 5 naps in multiple sleep latency test. SOL, sleep onset latency. TST, total sleep time. ^a^ The *p*-value was obtained using a chi-square test. ^b^ The *p*-value was obtained using two-sample t tests.

**Table 3:** Brain regions with different fALFF values in NT1 compared with controls.

Brain Regions	Abbr.	Side	Voxels	MNI Coordinates of Peak Point			Peak Intensity
				X	Y	Z	
NT1 > HCs							
Lingual gyrus	LING	R	381	15	−57	9	9.1208
Fusiform gyrus	FFG	L	227	−42	−72	−12	6.9931
		R	88	30	−75	−9	5.9668
Postcentral gyrus	PoCG	L	164	−51	−12	27	6.6821
Precentral gyrus	PreCG	R	162	36	3	33	7.3822
Inferior frontal gyrus, opercular part	IFGoperc	L	85	−48	12	−18	6.4628
Middle frontal gyrus	MFG	L	45	−30	63	−6	6.2985
NT1 < HCs							
Caudate nucleus	CAU	L	90	−3	9	0	−5.7451
Temporal pole, middle temporal gyrus	TPOmid	L	57	−45	6	−21	−5.3681
		R	43	42	18	−36	−4.5134
Parahippocampal gyrus	PHG	L	26	−30	3	−48	−4.3355
Precuneus	PCUN	L	17	0	−57	54	−4.9176
Anterior cingulate and paracingulate gyri	ACG	R	15	0	45	−18	−4.5432
Amygdala	AMYG	R	13	18	0	−18	−4.4282

fALFF, fractional low-frequency amplitude of low-frequency fluctuation. NT1, narcolepsy type 1. HCs, healthy controls. MNI, Montreal Neurological Institute. L, left. R, right.

**Table 4:** Brain regions with different FC in NT1 compared with controls.

Brain Regions	Abrr	Side	Voxels	MNI Coordinates of Peak Point			Peak Intensity
				X	Y	Z	
ROI 1: left temporal pole, middle temporal gyrus (TPOmid.L peak MNI −45 6 −21)							
NT1 > HCs							
Middle temporal gyrus	MTG	L	76	−42	6	−24	6.9776
Temporal pole, superior temporal gyrus	TPOsup	R	31	33	15	−27	5.0105
NT1 < HCs							
Postcentral gyrus	PoCG	R	754	48	−63	−12	−12.8433
Middle frontal gyrus	MFG	R	298	24	66	6	−6.3546
		L	242	−39	48	12	−5.7688
Lenticular nucleus, putamen	PUT	L	87	−21	24	−9	−5.6682
Superior temporal gyrus	STG	L	57	−42	−36	9	−5.1075
Inferior frontal gyrus, opercular part	IFGoperc	L	33	−48	15	21	−4.8033
Superior frontal gyrus, medial	SFGmed	L	33	−9	33	30	−5.6702
Supramarginal gyrus	SMG	R	28	54	−45	33	−5.0067
Insula	INS	R	28	51	0	6	−4.2619
Caudate nucleus	CAU	R	24	15	24	−3	−4.6738
**ROI 2: left caudate nucleus (CAU.L, peak MNI −3 9 0)**							
NT1 < HCs							
Fusiform gyrus	FFG	R	17	36	0	−48	−5.2097
**ROI 3: left precuneus (PCUN.L, peak MNI 0 −57 48 54)**							
NT1 > HCs							
Postcentral gyrus	PoCG	R	19	24	−45	6	4.7254
NT1 < HCs							
Precuneus	PCUN	R	74	−3	−63	39	−5.006
Superior frontal gyrus, medial	SFGmed	L	30	−15	54	30	−4.8208
Middle temporal gyrus	MTG	L	18	−39	−54	15	−4.5407

FC, functional connectivity. NT1, narcolepsy type 1. HCs, healthy controls. MNI, Montreal Neurological Institute. ROI, region of interest. L, left. R, right.

## Data Availability

All data is available from the authors upon reasonable request.
